# 7-Tesla Magnetic Resonance Imaging Precisely and Noninvasively Reflects Inflammation and Remodeling of the Skeletal Muscle in a Mouse Model of Antisynthetase Syndrome

**DOI:** 10.1155/2014/879703

**Published:** 2014-05-05

**Authors:** Clara Sciorati, Antonio Esposito, Lara Campana, Tamara Canu, Antonella Monno, Anna Palmisano, Francesco De Cobelli, Alessandro Del Maschio, Dana P. Ascheman, Angelo A. Manfredi, Patrizia Rovere-Querini

**Affiliations:** ^1^Division of Regenerative Medicine, Stem Cells & Gene Therapy, San Raffaele Scientific Institute, Via Olgettina 60, 20132 Milan, Italy; ^2^Department of Radiology and Preclinical Imaging Facility, San Raffaele Scientific Institute, Via Olgettina 60, 20132 Milan, Italy; ^3^School of Medicine, Vita-Salute San Raffaele University, Via Olgettina 58, Milan, Italy; ^4^University of Miami Miller School of Medicine, Miami, FL 33136, USA

## Abstract

Inflammatory myopathies comprise heterogeneous disorders. Their etiopathogenesis is poorly understood, because of the paucity of informative experimental models and of approaches for the noninvasive study of inflamed tissues. Magnetic resonance imaging (MRI) provides information about the state of the skeletal muscle that reflects various facets of inflammation and remodeling. This technique has been scarcely used in experimental models of inflammatory myopathies. We characterized the performance of MRI in a well-established mouse model of myositis and the antisynthetase syndrome, based on the immunization of wild-type mice with the amino-terminal fragment of histidyl-tRNA synthetase (HisRS). Over an eight-week period following myositis induction, MRI enabled precise identification of pathological events taking place in muscle tissue. Areas of edema and of active inflammation identified by histopathology paralleled muscle modifications detected noninvasively by MRI. Muscles changes were chronologically associated with the establishment of autoimmunity, as reflected by the development of anti-HisRS antibodies in the blood of immunized mice. MR imaging easily appreciated muscle damage and remodeling even if actual disruption of myofiber integrity (as assessed by serum concentrations of creatinine phosphokinase) was limited. Thus, MR imaging represents an informative and noninvasive analytical tool for studying *in vivo* immune-mediated muscle involvement.

## 1. Introduction

Inflammatory myopathies (IM) comprise a group of heterogeneous muscle diseases that share key common characteristics including in particular muscle weakness, low endurance [1], tissue infiltration by inflammatory cells [2–4], and myofiber necrosis/regeneration with an increase of creatine phosphokinase (CPK) serum levels during acute phases of the disease [5]. The presence of autoantibodies targeting ubiquitous intracellular proteins involved in gene transcription or protein synthesis and translocation [6] highlights an autoimmune origin of the disease. Autoantibodies against histidyl-tRNA synthetase (HisRS, also called Jo-1) are particularly well-studied [7, 8], and their serum level correlates with various measures of disease activity [9].

The pathogenesis of IM is jet poorly understood. Animal models that fully reproduce the various features of human disease are needed [10]. Myositis induced upon immunization with HisRS appears particularly informative, since it reproduces both the break of tolerance towards selected autoantigens and specific combined inflammatory involvement of the skeletal muscle and lung that are hallmarks of the human antisynthetase syndrome [11, 12]. Despite the insight provided by such models, the application of noninvasive methods for* in vivo* monitoring of disease activity, such as magnetic resonance imaging (MRI), has been lacking.

Magnetic resonance imaging (MRI) is a powerful and informative technique to investigate soft tissues, with the ability to noninvasively characterize parenchymal changes occurring in patients with myositis. Imaging has traditionally had an ancillary role in the diagnosis of myositis and inflammatory myopathies. Routinely, the MRI protocol includes T1-weighted images and fluid-sensitive sequences such as short tau inversion recovery (STIR), providing qualitative information about muscle tone, fat infiltration, and muscle edema [13]. Transaxial and coronal sections of the shoulders and thighs are usually obtained on each patient. T1-weighted images allow to assess the muscle thickness/mass and to score the degree of fatty infiltration; fluid-sensitive sequences detect the presence of edema [13]. Routine MRI performed with T1-weighted and STIR sequences is more sensitive but less specific than biopsy in diagnosis; it is advantageous for optimizing efficacy of classical diagnostic procedures [14]. Its importance is progressively growing, since it allows to noninvasively characterize the distribution and pattern of parenchymal changes and to monitor the disease progression, which has important implications for treatment [13, 15].

In the mouse, MRI has been used after tissue injury induced by maximal lengthening contractions [16] and in experimental models of skeletal muscle dystrophy (*mdx* and dysferlin-deficient mice) to assess disease progression [17]. Foci of high intensity signal in T2-weighted images correspond to dystrophic lesions in* mdx* mice [18, 19], while changes in gadofluorine enhancement were identified in dysferlin-deficient animals [20]. Recently, MRI has also been used in C57BL/6 mice to assess the specific features of the homeostatic response of healthy muscles to acute sterile injury induced by cardiotoxin (CTX) [21]. Specifically, T2 mapping and diffusion-tensor imaging (DTI) provide useful information on the extent of myofibril necrosis and of leukocyte infiltration as well as on the kinetics of regeneration [21]. Here we show that MRI, including advanced quantitative techniques as T2 mapping and DTI, is a useful tool to assess the inflammatory changes and the tissue remodeling associated with autoimmune myositis in an experimental model of HisRS-induced myositis. Architectural changes in the tissue organization were temporally linked to muscle damage and to the establishment of the specific autoantibody response.

## 2. Materials and Methods

### 2.1. Animal Model and Study Design

Sterile injury by CTX injection and immunization experiments was carried out using C57BL/6J wild-type mice (Charles River, Wilmington, MA, USA) aged 8–10 weeks. Animals were housed in the pathogen-free facility at our institution and treated in accordance with the European Community guidelines and with the approval of the Institutional Ethical Committee (IACUC number 512). For sterile injury, animals were anesthetized by intraperitoneal injection of tribromoethanol (Avertin) at a dose of 250 mg/Kg and* tibialis anterior* (TA) muscles were injected with CTX (50 *μ*L, 10 *μ*M final concentration,* Naja mossambica mossambica*, Sigma-Aldrich) in phosphate buffer solution (PBS) using an insulin needle (3/10 cc Insulin Syringe from Becton-Dickinson, Franklin Lakes, NJ, USA). Vehicle-treated mice received PBS alone (50 *μ*L). For HisRS immunization,* gastrocnemius* (GS) muscles were injected with a recombinant protein fragment corresponding to the amino-terminal portion (residues 1–151) of the murine HisRS molecule produced as a maltose binding protein and characterized as described in [11]. Immunization was carried out using a mixture of the antigen (4 mg/mL) and incomplete Freund's adjuvant (IFA; 1 : 1, vol : vol; 100 *μ*L/mouse). Vehicle-treated mice received a mixture of PBS and IFA. Independent cohorts (6 mice/group) were monitored by MRI after myositis induction, sham treatment (IFA/PBS), or sterile CTX injury at various time points: times 0 and 7, 14, 28, or 56 days after HisRS immunization and times 0, 1, 3, 7, 10, 15, and 30 days after CTX injection.  Table 1 shows experimental timing for the two different models of damage.

### 2.2. MRI

MRI studies were performed on a 7T preclinical magnetic resonance scanner (Bruker, BioSpec 70/30 USR, Paravision 5.0, Germany), equipped with 450/675 mT/m gradients (slow rate: 3400–4500 T/m/s; rise time: 140 *μ*s). A phased-array rat-heart coil with four internal preamplifiers was used as receiver, coupled with a 72 mm linear-volume coil as transmitter. Mice were under general anesthesia obtained by 1,5–2% isoflurane (Forane, Abbott) vaporized in 100% oxygen (flow: 1 l/min), in prone position, with the right leg fixed in the center of the coil. Breathing and body temperature were monitored during MRI (SA Instruments, Inc., Stony Brook, NY, USA) and maintained around 30 breaths per minute and 37°C, respectively. After positioning in the magnet isocenter, a fieldmap based shimming (MAPSHIM software package, Paravision-5.0, Bruker; Germany) was performed to optimize B0 field homogeneity. MRI parameters were used to assess skeletal muscle that included T2-relaxation time (T2-rt), evaluated by T2 mapping, and fractional anisotropy (F.A.), evaluated by diffusion tensor imaging (DTI). Muscle T2 maps were obtained using a multislice-multiecho (MSME) sequence with fat suppression (repetition time = 1938 ms; 16 echo times = 10.73/171.68 ms; field-of-view = 20 × 20 mm; matrix = 256 × 256; spatial resolution = 0.078 × 0.078 mm/pixel; NSA = 4) acquired on axial plane (10 slices; thickness = 1 mm; gap = 0 mm). DTI data were obtained using a SpinEcho-EPI sequence (DTI-Epi) with 30 diffusion gradient directions (repetition time = 3750 ms; echo time = 33 ms;* b*-values for direction = 0 sec/mm^2^–700 sec/mm^2^; diffusion gradient duration = 4 ms; diffusion gradient separation = 20 ms; NSA = 2). DTI-Epi sequence shared the same geometrical features (field-of-view = 30 × 30 mm; matrix = 128 × 128; spatial resolution = 0.234 × 0.234 mm/pixel; 10 slices; slice thickness = 1 mm; gap = 0 mm).

### 2.3. Image Analysis

MRI postprocessing was performed with Paravision-5.0 software (Bruker). Average ADC, F.A., and T2-rt values were obtained from the regions of interest (ROIs) of five subsequent slices placed both on TA (CTX-injured muscle) and on GS (HIsRS-injected muscle) muscles of each mouse at each time point.

### 2.4. Autoantibodies

Anti-HisRS IgG antibodies in the serum were measured using standard solid-phase enzyme-linked immunosorbent assay (ELISA) as described [11, 12]. Briefly ninety-six-well plates (Sigma) were coated with recombinant murine synthetic HisRS N-terminal fragment sequence (0.1 *μ*g/mL) and incubated overnight at 4°C. After blocking with PBS containing 1% bovine serum albumin (BSA), serum at various dilutions in PBS containing 1% BSA was added for 2 hours. Following incubation with HRP-conjugated goat anti-mouse IgG (Sigma), the enzymatic reaction was visualized using 3,3′,5,5′-tetramethylbenzidine (Sigma) and terminated by H_2_SO_4_ addiction prior to spectrophotometric assessment at 450 nm using a microplate reader (Biorad, Hercules, CA) [11, 12].

### 2.5. Histological Analysis and Image Acquisition

Animals were sacrificed by cervical dislocation and muscles were dissected, immediately frozen in liquid N_2_-cooled isopentane, and conserved at −80°C until analysis. For histological studies, 8 *μ*m serial muscle sections were obtained and stained in hematoxylin and eosin (H&E) following standard procedures. At least 10 sections along the entire muscle length were collected and stained. An expert blinded pathologist evaluated samples assigning a 0-to-5 score (0: healthy muscle and 5: maximum inflammatory infiltration), based on the degree of inflammatory cells identified. For image acquisition, we employed a microscope Nikon Eclipse 55i microscope (Nikon, Tokyo, Japan). Images were captured with Digital Sight DS-5 M digital camera (Nikon) using Lucia G software (Laboratory Imaging, Prague, Czech). Linear adjustments of images were done using Adobe Photoshop CS4.

### 2.6. CPK Activity

Serum CPK levels were measured in blood samples obtained from the tail vein using an indirect commercially available colorimetric assay according to the manufacturer's instructions (Randox, UK).

### 2.7. Statistical Analysis

All values were expressed as mean ± S.E.M. All data were analyzed using an unpaired two-tailed Student's* t*-test for comparisons between two groups. *P* values lower than 5% (*P* < 0.05) were considered statistically significant.

## 3. Results

**Figure 1:**
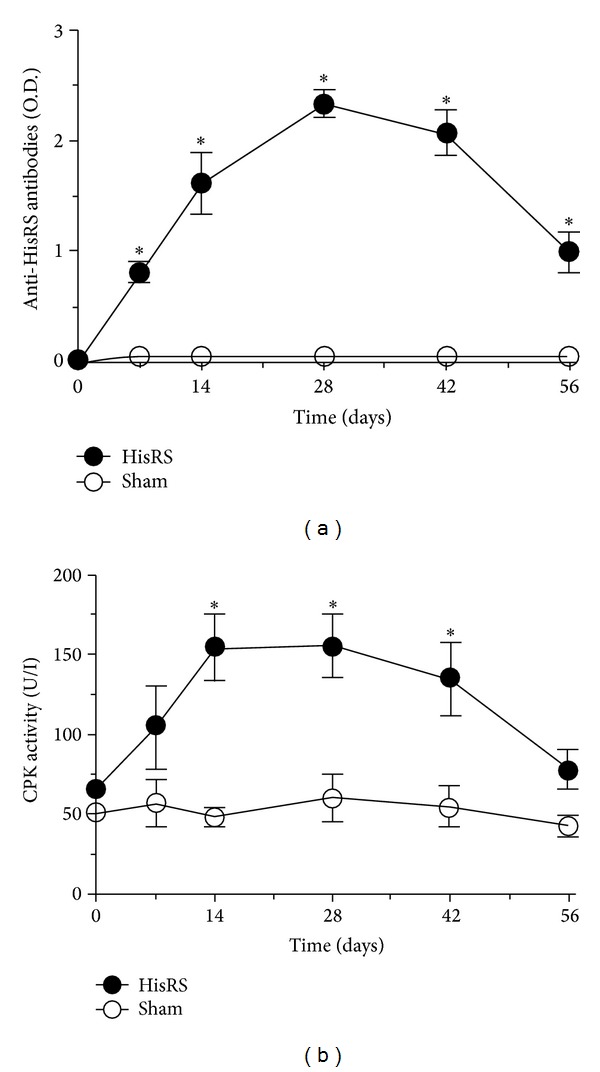
*Anti-HisRS autoantibody induction is associated with sustained muscle damage.* Assessment of anti-HisRS IgG antibodies (a) and CPK activity (b) in serum retrieved before treatment and at various times after immunization. *n* ≥ 6;  *statistically different from sham-treated mice; *P* < 0.05.

We analyzed changes in the normal architecture of mouse skeletal muscle by multiparametric MRI comparing features associated with the homeostatic response to sterile tissue injury with those associated with the immune-mediated skeletal muscle remodeling characterizing a model of antigen-induced experimental myositis. 10-week-old wild-type mice were injected in GS muscle at day 0 with a synthetic sequence corresponding to the amino-terminal portion of the murine HisRS autoantigen in the presence of adjuvant (IFA). Mice injected with IFA alone (sham-treated) served as controls. Autoantibody induction, CPK levels, histopathological abnormalities, and imaging evidence of muscle inflammation/remodeling were verified at various time points (see Table 1). Anti-HisRS IgG antibodies were detectable by seven days after immunization and increased progressively until day 28, at which point their levels began to decrease. (Figure 1(a)). Two months after a single immunization, antibodies were still significantly higher than before treatment or than in sham-treated mice that never develop detectable anti-HisRS antibodies. Autoimmunity establishment and production of autoantibodies correlate with myofibers necrosis, as assessed by the serum CPK activity which reflects the disruption of sarcolemma integrity: in fact CPK levels were significantly higher in immunized mice starting from two weeks to six weeks after immunization. CPK levels in sham-treated animals remained at background levels, indicating that intramuscular injection per se is not responsible for enzyme elevation (Figure 1(b)).

**Figure 2:**
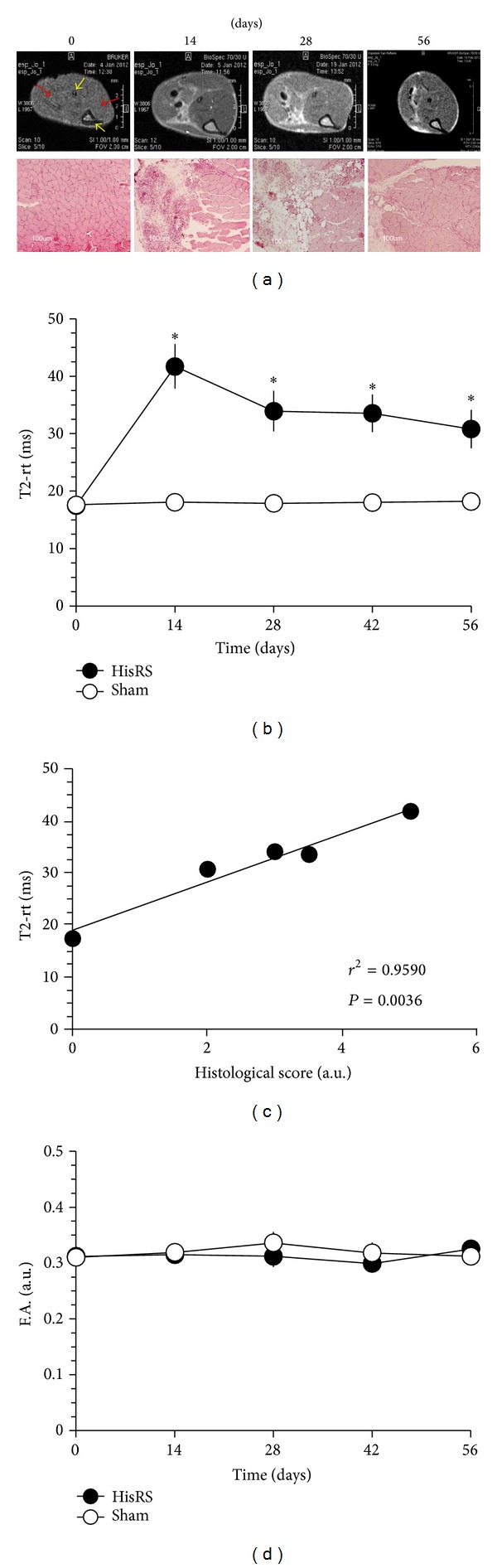
*T2 MRI mapping reflects inflammation and remodeling in experimental HisRS-induced myositis*. Representative T2-weighted images of immunized mice ((a) upper panels) obtained before (time 0) and 14, 28, or 56 days after treatment reveal ongoing inflammatory involvement and are well compatible with results obtained at the same time points by histopathology in selected animals ((a) lower panels, H&E). Yellow arrows indicate the fibula (upper) and tibia (lower) bones, while red arrows show skeletal muscles (GS left and TA right). The graphs show the dynamic trends of T2-rt ((b)* y*-axis) and F.A. ((d)* y*-axis) in the longitudinal study of immunized and sham-treated mice before and at various times after treatment (*x*-axis, days). *n* ≥ 6;  *statistically different from sham-treated mice; *P* < 0.05. The graph in (c) depicts correlation analysis between T2-rt and the histological score that reflects muscle damage and infiltration (see Methods). *n* = 3 for each time point. The time points considered for the correlation analysis were days 0, 14, 28, 42, and 56.

In animal cohorts studied by the noninvasive technique of MRI, tibial and peroneal bones can be easily identified in noninjured healthy wild-type animal legs (Figure 2(a), yellow arrows) and provide anatomical landmarks for location of TA and GS muscles. Skeletal muscle has intermediate signal intensity on all MR pulse sequences, with T2-relaxation time images (T2-rt) that are rather homogeneous (Figure 2(a), red arrows).

**Figure 3:**
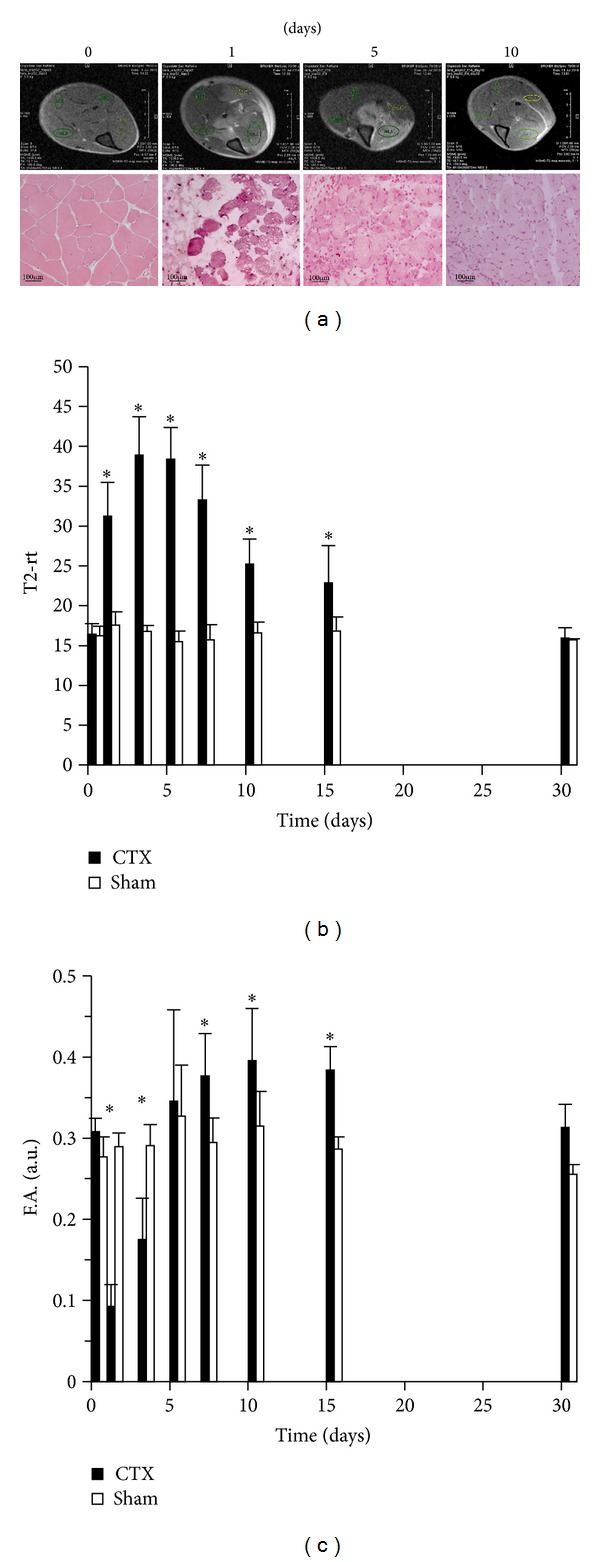
*T2 and DTI MRI mapping reflect independent inflammatory events in acutely injured muscles.* Representative T2-weighted images before (time 0) and at various times (1, 5, or 10 days) after intramuscular CTX injection ((a) upper images). Results are compatible with those obtained at the same time points by histopathology in selected animals ((a) lower panels, H&E). The graphs show the dynamic trends of T2-rt ((b)* y*-axis) and F.A. ((c)* y*-axis) in the longitudinal study of damaged (CTX) and sham-treated mice before and at various times after treatment (*x*-axis, days). *n* ≥ 6;  *statistically different from sham-treated mice; *P* < 0.05.

Muscles of immunized mice demonstrated a significant increase in the T2-rt values by two weeks after immunization (Figure 2(b)), that is, at a time in which CPK levels revealed ongoing myofibers necrosis (Figure 1(b)). T2-rt values remained significantly higher throughout the observation period that lasted until 8 weeks after immunization. Interestingly, and in sharp contrast with the characteristics of the T2 mapping in acutely injured muscles (see Figure 3), alterations detected by MRI appeared to be restricted to the regions at the very periphery of leg muscles, preferentially involving the perimuscular connective tissues (Figure 2(a)). In contrast, T2-rt values of sham-treated mice remained very stable after intramuscular injection. Of interest, DTI assessment revealed that F.A. values were substantially stable after immunization and did not differ from those of sham-treated animals (Figure 2(d)). We then verified the histological correlates of the results, observing that tissues of immunized animals undergo a preferential fragmentation of perimysial connective tissue, which is characterized from intense infiltration by mononuclear cells. Edema with limited evidence of necrosis and myofiber atrophy most frequently occurred in proximity to inflamed connective tissue. In contrast, inflammatory involvement of endomysial and perivascular regions was uncommon, with only some degree of muscle regeneration. (Figure 2(a)). Extending these observations, we assessed the link between T2-rt values of immunized mice at various time points and a tissue histological score for inflammation (see the Materials and Methods section for details). T2-rt values and the histological damage/infiltration score were significantly correlated (*r* = 0.959; *P* < 0.005, Figure 2(c)), suggesting that T2-rt values accurately reflect perimysial inflammation.

As an internal control for ongoing muscle remodeling, we studied in parallel the homeostatic response to sterile injury by CTX, an agent that induces transient muscle necrosis associated with well-characterized reversible alterations detectable by multiparametric MRI ([21] and Figure 3). T2-rt values 1 day after sterile injury were significantly higher than those of sham-treated mice and similar to those observed in mice with autoimmune myositis at later time points (compare Figure 3(b) with Figure 2(b)). Kinetic was remarkably different, since in CTX-injured mice T2-rt values dropped to background levels ten days after treatment and remained stable thereafter (Figure 3(b)). Parallel histopathological study of muscle tissue obtained from mice sacrificed after imaging revealed that damage resulted in rapid and massive death of myofibers by day 1, with subsequent infiltration of the tissue by inflammatory cells and appearance of small regenerating/centronucleated fibers by day 5 after injection. At day 10, necrosis and inflammation were hardly apparent, while extensive regeneration was taking place (Figure 3(a)). In contrast, F.A. values dropped 24 hours after injury, possibly because of the simultaneous necrosis of myofibers that abruptly lose their architecture in response to CTX (Figure 3(c)). With time, F.A. values returned to normal in conjunction with the successful regeneration of the tissue. Similarly, F.A. values remained relatively stable in HRS-induced myositis, that is, associated with fairly limited necrosis and regeneration (Figure 2(d)), further supporting the relationship between F.A. values and the state of muscle repair.

## 4. Discussion 

IM are heterogeneous disorders, histologically characterized by muscle inflammation with the presence of autoreactive lymphocytes, fiber degeneration, and overexpression of MHC class I. Common clinical features are muscle weakness, serum autoantibodies, and elevated muscle enzyme [7, 8]. Recent data suggest not only that both innate immunity and adaptive immunity are involved in the IM pathogenesis but also that intrinsic muscle defects (such as metabolic defects and endoplasmic reticulum stress and autophagy and hypoxia) may contribute to damage [1]. The link between remodeling of skeletal muscle and autoimmune responses, which typically target ubiquitous expressed autoantigens, is extremely difficult to study, as the events underlying reduced muscle strength and endurance [8, 22]. The availability of informative animal models is thus crucial [23]. Traditionally, the assessment of ongoing muscle inflammatory involvement has been rather cumbersome, since by definition it requires histopathological studies and animal sacrifice.

While replacement is difficult to achieve in this field of research, given the complexity of events involved in myositis pathogenesis that can be only reproduced in* in vivo* systems, the availability of noninvasive tools to monitor muscle inflammation will permit reductions in the number of animals necessary for experimental studies and increase the amount of relevant information that can be derived from single animals followed over time. At the same time, this imaging modality will complement information derived from histopathological studies that encompass only limited areas of tissue and therefore suffer from potential sampling bias.

Here, we have focused on MRI-based imaging techniques that have been previously shown to represent noninvasive and quantitative tools for the assessment of muscle inflammation and remodeling in experimental animals [13, 16, 21, 24, 25]. We utilized a biologically relevant model of experimental myositis, which can be elicited by immunization of wild-type mice with a synthetic sequence corresponding to a well-characterized autoantigen, HisRS [11, 12]. This model triggers anti-HisRS autoantibodies as well as self-sustaining injury and leukocyte infiltration of skeletal muscle, reproducing key features of spontaneous human disease that can also be associated with fever, arthritis, mechanic's hands, Raynaud's phenomenon, and interstitial lung disease as part of the antisynthetase syndrome [26]. Reference [27] shows that muscle inflammation has specific features in these patients with the antisynthetase syndrome, with predominant involvement of perimysial connective tissue and less significant peripheral atrophy or microvascular injury [28, 29].

As shown by our results, MRI is a powerful tool to investigate muscle inflammation occurring in the mouse model of HisRS-induced myositis. Most significantly, cohorts of mice can be longitudinally followed with this technique that accurately detects specific histopathological features of the events taking place in the tissue upon autoimmunity. In our study, MRI proved extremely sensitive, detecting early tissue changes associated with relatively minor increases in the extent of muscle necrosis revealed by changes in serum CPK levels. Importantly, MRI of sham-treated animals remained stable over the 8 weeks of the study, effectively ruling out major effects related to the immunization vehicle, IFA.

Overall, our study provides strong evidence of the link between HisRS-induced immune responses and myositis that occurs, as early as fourteen days after immunization. Moreover, noninvasive T2 mapping reveals the involvement of perimysial connective tissue, that is, a specific feature of the human antisynthetase syndrome [28], clearly differentiating HisRS-induced myositis from the CTX model of acute, self-limited muscle necrosis. As such, the T2-rt values effectively mirror the extent and distribution of muscle inflammation, not only in the setting of homeostatic responses to sterile toxic injury [21], but also in a more complex and long-lasting model of immune-mediated muscle remodeling. Of importance, the degree of anisotropy of water diffusivity remained substantially stable in the mice that we have studied upon myositis induction, contrasting sharply with MRI results obtained in CTX-treated mice. Because this parameter largely reflects the diffusion of water in intracellular spaces [24, 30], these results are in agreement with the preferential involvement of the perimysial tissue (reflected by clear histological evidence of edema in the perifascicular space) and the substantial conservation of tissue architecture in HisRS-induced myositis.

Based on these observations, our findings indicate that MRI represents a versatile tool that can sensitively detect inflammatory processes associated with various types of muscle injury. At the same time, this imaging modality affords the opportunity to noninvasively monitor more subtle modifications in the architecture and inflammatory state of skeletal muscle, including those changes associated with initial age related decline in muscle mass and simultaneous increases of both T2-rt and F.A. Further studies are warranted to verify whether specific MRI codes are also associated with other conditions characterized by persistent inflammation and wasting of the skeletal muscle.

## 5. Conclusions

In conclusion, our study was designed to characterize the ability of MR imaging to reveal specific inflammatory events in the skeletal muscle of mice with HisRS-induced myositis. Our results provide an in-depth noninvasive characterization of this myositis model, confirming the possibility to link HisRS-induced immune responses and skeletal muscle inflammation to MRI T2-rt changes. This technique allows increasing the amount of relevant information that can be derived from single animals followed over time and may strongly improve and facilitate the study of new therapeutic strategies for human disease as well as of the immunological and local events involved in myositis pathogenesis. At the same time, this imaging modality can help histopathological studies encompassing their potential sampling bias with complement quantitative information. The possibility to follow the same animal from the beginning to the end of treatment and to analyze more parameters at ones, can allow better statistically evaluation of data and underlining the concept that strong efforts should be dedicated to the refinement of experimental procedures that limit the number of animals needed for experimental models of human disease.

## Figures and Tables

**Table 1:** Experimental settings and timing of evaluations.

	CPK and autoantibodies	MRI	Histopathology
Autoimmunity (HisRS immunization)	0, 7, 14, 28, 42, 56	0, 7, 14, 28, 42, 56	0, 7, 14, 28, 56
Sterile injury (CTX injection)		0, 1, 3, 5, 7, 10, 15, 30	0, 1, 3, 5, 10, 30
